# Clinical Characteristics and Special Considerations in the Management of Rare Melanoma Subtypes

**DOI:** 10.3390/cancers16132395

**Published:** 2024-06-28

**Authors:** Adrienne B. Shannon, Jonathan S. Zager, Matthew C. Perez

**Affiliations:** Department of Cutaneous Oncology, H. Lee Moffitt Cancer Center & Research Institute, Tampa, FL 33612, USA; adrienne.shannon@moffitt.org (A.B.S.); jonathan.zager@moffitt.org (J.S.Z.)

**Keywords:** acral melanoma, mucosal melanoma, uveal melanoma, desmoplastic melanoma, advanced melanoma, atypical melanoma, atypical skin cancer

## Abstract

**Simple Summary:**

Advancements in immune, targeted, and regional therapies have emerged in the past fifteen years in the management of high-risk, advanced, and metastatic melanomas. Rare melanoma variants include acral, mucosal, uveal, and desmoplastic melanoma. Though underrepresented in larger landmark trials for modern therapeutic options, results from these trials have been extrapolated and applied in the management of these rarer subtypes of melanoma. This article aims to review the current treatment recommendations for acral, mucosal, uveal, and desmoplastic melanomas.

**Abstract:**

Rare histologic subtypes of melanoma, including acral, mucosal, uveal, and desmoplastic melanomas, only make up 5% of all diagnosed melanomas and are often underrepresented in large, randomized trials. Recent advancements in systemic therapy have shown marked improvement in pathologic response rates, improving progression-free and overall survival among cutaneous melanoma patients, but there are limited data to demonstrate improved survival among rarer subtypes of melanoma. Acral melanoma has a poor response to immunotherapy and is associated with worse survival. Mucosal melanoma has a large variability in its presentation, a poor prognosis, and a low mutational burden. Uveal melanoma is associated with a high rate of liver metastasis; recent adoption of infusion and perfusion therapies has demonstrated improved survival among these patients. Desmoplastic melanoma, a high-risk cutaneous melanoma, is associated with high locoregional recurrence rates and mutational burden, suggesting this melanoma may have enhanced response to immunotherapy. While these variants of melanoma represent distinct disease entities, this review highlights the clinicopathologic characteristics and treatment recommendations for each of these rare melanomas and highlights the utility of modern therapies for each of them.

## 1. Introduction

Melanoma is the fifth most common cancer in the United States, with an annual increased incidence of 1.8% for males and 2.5% for females [1]. Despite significant improvements in the survival of melanoma over the past two decades, melanoma still represents the vast majority of deaths related to skin cancers. Rare histologic subtypes of melanoma, such as acral, mucosal, uveal, and desmoplastic melanomas, comprise up to 5% of all diagnosed melanomas and are often underrepresented in landmark trials.

Improvements in survival outcomes among melanoma patients over the last 15 years are attributable to pharmacologic advancements. Systemic therapy for patients with advanced melanoma has been revolutionized with the evolution of immune checkpoint inhibitors. Following Food and Drug Administration (FDA) approval of the anti-cytotoxic T-lymphocyte-associated protein 4 (CTLA-4) agent ipilimumab in 2011, studies noted an objective response rate (ORR) of 15% among patients with advanced stage III or stage IV disease [2]. Subsequent studies evaluating the use of anti-programmed death-1 (anti-PD-1) agents noted a complete response rate of 16% and an ORR of 30–40% among similar populations of patients. Furthermore, combined therapies, including anti-CTLA-4 and anti-PD-1 agents, have been noted to result in an ORR of 50–60% and a complete response rate of 19–22% in this population, changing the prognosis associated with this disease [2,3,4,5]. More recently, a phase II randomized trial examining perioperative (neoadjuvant and adjuvant) use of pembrolizumab as compared to adjuvant pembrolizumab alone in patients with resectable stage III/IV melanoma demonstrated longer event-free survival (72% vs. 49% at 2 years of follow-up) among patients within the neoadjuvant–adjuvant treatment arm [6].

Given the sparse incidence of rare subtypes of melanoma, these tumors are often underrepresented in the larger landmark trials that dictate the current treatment recommendations for advanced melanoma. Therefore, the management of these rare histologic subtypes of melanoma is largely secondary to the extrapolation of data from these larger studies, but this practice does not always reflect and capture the distinct clinical, genetic, and histopathologic differences among these tumors. This review serves to highlight the unique clinical features and available evidence regarding treatment response for patients with acral, mucosal, uveal, and desmoplastic melanomas.

## 2. Acral Melanoma

### 2.1. Epidemiology

Acral lentiginous melanoma is a rare subtype of melanoma that arises on glabrous, or non-hair bearing, skin, including the nailbeds, palms, and soles of the feet, and is associated with an overall worse prognosis when compared to non-acral cutaneous melanoma [7,8,9,10,11,12]. Acral melanoma makes up 2–3% of all diagnosed melanomas [7,13]. While the incidence of acral melanoma is similar across racial and ethnicity groups, acral melanoma represents 33–36% of melanoma diagnoses in Black patients, 18–23% of melanoma diagnoses in Asian and Pacific Islander patients, and only 1% of melanoma diagnoses in non-Hispanic White patients [7]. Incidence rates of acral melanoma in the United States based on Surveillance, Epidemiology, and End Result (SEER) registry studies show an overall incidence of 1.8 per 1,000,000 persons; this incidence is highest among Hispanic White patients at 2.8 per 1,000,000 persons and lowest among Asian and Pacific Islander patients at 1.5 per 1,000,000 persons [7,13]. In countries outside of the United States, acral melanoma represents as much as 50–60% of Asian patients with melanoma and up to 80% of African patients with melanoma [14,15,16,17].

### 2.2. Presentation and Histopathology

The etiology of acral melanoma is poorly understood, particularly given that ultraviolet (UV) sun exposure and fair skin type, risk factors for other traditional cutaneous melanoma subtypes, do not appear to correlate with the development of acral melanoma. Acral melanoma most often has an early asymmetrical, macular appearance with variable pigmentation. Subungual lesions are a specific group of acral melanoma found within the nailbeds and may have partial or total melanonychia of the nailbed in connection with periungual skin pigmentation, known as Hutchinson’s sign. On dermoscopic evaluation, acral melanomas often have a parallel ridge pattern that distinguishes them from an acral nevus [18]. Histologically, acral melanoma features include a radial growth pattern of pagetoid melanocytes associated with dendritic morphology and extension into the sweat gland epithelium; fibrillar and parallel furrowing patterns would be more consistent with an acral nevus [19]. Less than 10% of lesions are associated with a pre-existing acral nevus, and congruent with the lack of correlation between UV sun exposure and acral melanoma, most patients have a lower solar erythema rate than is seen with traditional cutaneous melanoma patients [8,20]. Additionally, acral melanoma is associated with a low number of point mutations and a high rate of copy number variations, resulting in amplification of genes CCDN1 and KIT and TERT translocations [21].

Most lesions (66%) arise on the plantar surfaces, but 17% occur on the subungual finger, 12% occur on the subungual toe, and 5% occur on the palmar surfaces [8]. Acral melanoma is often misdiagnosed as a benign entity, such as diabetic foot ulcers, traumatic-related hemorrhage (or talon noir), fungal and bacterial infections, and verrucae, leading to advanced presentation at the time of diagnosis. If there is suspicion of acral melanoma, particularly if treatment for a presumed benign condition appears ineffective, it is recommended that biopsies be performed on the acral skin as well as the nailbed if indicated. Appropriate biopsy techniques for these lesions include punch biopsy, lateral longitudinal excision, and shave biopsy. However, these lesions are technically often difficult to biopsy, resulting in incomplete biopsies. In a review of 71 patients with partially sampled acral and non-acral melanocytic lesions, 18.3% and 8.5% of patients were upstaged on further sampling of the residual lesion to either undergo sentinel lymph node biopsy or wider local excisional margins, respectively [22,23].

**Figure 1:**
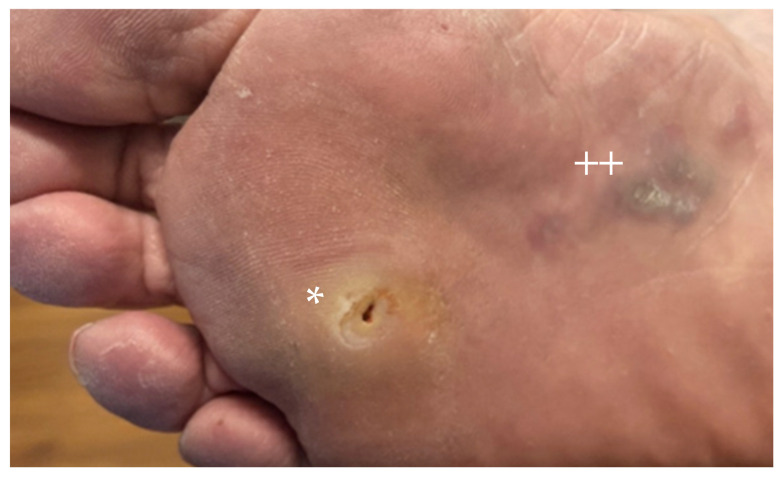
Primary plantar foot acral melanoma (*) shown with adjacent area of in-transit disease (++).

In general, survival outcomes are worse among acral melanoma patients as compared to non-acral cutaneous melanoma patients [7,8,9,10,11,12,24,25]. Acral melanoma often presents at a more advanced stage (Figure 1); this was previously believed to be secondary to a delay in diagnosis, but some studies have suggested a worse prognosis even when controlling for the advanced stage [19,24]. Negative prognostic factors for survival that are noted in the literature include thickness ≥1.5 mm, Clark’s level IV, and non-White race, though some have suggested the latter is a socioeconomic factor that may lead to delay in diagnosis [8]. Locoregional recurrence is more common among acral melanoma (39%) as compared to non-acral melanoma (19%), with a median recurrence-free survival in acral melanoma of 47 months as compared to 213 months in non-acral melanoma patients [24]. Despite this, this study quoted no difference in the rate of distant recurrence between these two entities (16% vs. 15%, *p* = 0.84) [24].

### 2.3. Treatment for Localized Disease

The standard of care for localized cutaneous melanoma, including acral subtypes, is a complete wide excision with margins of 1 to 2 cm, depending on the maximal depth of the tumor and in accordance with the current National Comprehensive Cancer Network (NCCN) guidelines based on multiple randomized controlled trials [26,27,28,29,30,31,32,33,34]. As an underrepresented subtype within these larger trials, the role of larger margins in acral melanoma is not defined, and often, the location of acral melanomas limits the feasibility of wound closure. Patients undergoing excision may require complex wound reconstruction with the use of skin grafting and flap reconstruction to help facilitate wound closure. Historically, amputation has been used for the definitive management of subungual lesions, yet the recent literature has suggested that non-amputative wide excision, when feasible, may be sufficient for the management of early-stage local disease [8,35].

The role of sentinel lymph node biopsy in patients with cutaneous melanoma, including acral melanoma, follows current NCCN guidelines that use primary tumor depth and ulceration status to guide recommendations. Yet, acral melanoma is associated with higher rates of sentinel lymph node positivity, with an overall positivity rate of 25.7% across stages, suggesting the important role of this procedure at the time of local excision [36]. Given this high positivity rate, in addition to the high rate of upstaging primary tumors after definitive excision, it is reasonable to consider sentinel lymph node biopsy in patients with T1a lesions and residual tumor and/or pigmentation after biopsy.

### 2.4. Advanced Disease

Given the limited representation of acral melanoma in landmark trials, there are limited data to suggest the efficacy of systemic immune and targeted therapies, specifically in the acral melanoma subtype. Acral melanoma typically has a lower mutation burden and lower rates of B-RAF proto-oncogene (BRAF) and neuroblastoma RAS viral oncogene homolog (NRAS) mutations as compared to its non-acral melanoma counterparts. In BRAF V600E-mutant acral melanoma, genomic sequencing has indicated that these lesions behave more like non-acral cutaneous melanoma as they lose some of the gene amplifications often noted in acral melanomas [37,38,39]. Bai and colleagues conducted a retrospective analysis in which 28 patients had BRAF V600E-mutated metastatic acral melanoma and an ORR of 38.1% following treatment with BRAF-targeted therapy, suggesting a lower response rate than those reported with BRAF-mutant non-acral cutaneous melanoma [40].

Given the lower tumor mutation burden found in acral melanomas, these lesions may be less responsive to immunotherapy [41,42]. In early studies evaluating the efficacy of immune checkpoint inhibitors, the ORR was lower for acral melanomas. In those with advanced acral melanomas, following the receipt of first-line treatment with ipilimumab, the ORR is as low as 17.8%, with a median progression-free survival (PFS) of 6.9 months [43]. Subsequent studies have shown that anti-PD-1 therapy monotherapy yields improved clinical responses as compared to ipilimumab monotherapy [44]. The KEYNOTE-151 trial, though not designed to evaluate results specifically in acral melanoma, was a phase Ib study in China evaluating the anti-programmed death 1 (PD-1) agent pembrolizumab as second-line therapy in advanced or metastatic melanoma. In this study, the ORR was 15.8% in acral melanoma (*N* = 39) patients [45]. The CheckMate-172 trial was a phase II multicenter study that stratified rare subtypes of advanced melanoma treated with nivolumab after having disease progression while on ipilimumab. Within the trial’s cohort, 55 (5.5%) patients had acral melanoma. The primary endpoint was the incidence of grade III treatment-related adverse events; there was no difference noted among patients with acral melanoma as compared to the larger melanoma population [46]. Median OS, a secondary endpoint, was 25.8 months among acral melanomas, similar to the median OS of non-acral cutaneous melanoma patients (25.3 months) [9].

Shoushtari and colleagues performed a retrospective multicenter study examining 25 patients with advanced acral melanoma who were treated with anti-PD-1 agents, with the majority of patients (85%) having received prior systemic therapy, primarily ipilimumab [42]. Among these patients, the ORR was 32%, and the median progression-free survival (PFS) was 4.1 months. An additional retrospective study performed by Nakamura and colleagues in Japan noted that, among 193 patients with advanced acral melanoma who received nivolumab or pembrolizumab, the ORR was 16.6%, and the median OS was 18.1 months [47]. However, the ORR for patients with lesions on the palm or sole (*N* = 123) was 21.1%, whereas the ORR for patients with lesions of the nail apparatus (*N* = 70) was 8.6% (*p* = 0.03). The median OS was 22.3 months and 12.8 months, respectively, mirroring this difference in response [47]. More recently, a phase II trial examining the use of apatinib, a tyrosine kinase inhibitor, in combination with camrelizumab, an anti-PD-1 inhibitor agent, in treatment-naïve advanced acral melanoma has demonstrated a disease control rate (DCR) of 82%, ORR of 24.1%, and median PFS and OS of 7.4 and 13.4 months, respectively [48].

There has been much debate about the utility of combined immune checkpoint inhibition in advanced acral melanomas, given the decreased response rates seen with single-agent therapy when compared with non-acral cutaneous melanoma. Bhave and colleagues conducted a multicenter retrospective study analyzing 325 patients with unresectable stage III or stage IV acral melanoma treated with a combination of anti-PD-1 therapy and ipilimumab as compared to monotherapy with either ipilimumab or anti-PD-1 monotherapy [41]. The ORR was higher in patients treated with combination immunotherapy as compared to anti-PD-1 agents alone (43% vs. 26%) and ipilimumab alone (43% vs. 15%). Despite this, there was no statistically significant difference in OS or PFS [41]. Additionally, Nakamura and colleagues examined this question in a multicenter Japanese study of 254 patients with unresectable stage III/IV acral melanoma treated with either combination immunotherapy (anti-PD-1 and ipilimumab) or anti-PD-1 therapy alone. There was no difference in ORR between the treatment arms when examining a sub-group of patients with palm and sole acral melanomas, but there was a significant improvement in ORR among patients with subungual melanomas who were treated with combination immunotherapy (61% vs. 10%) [49].

More recently, Tawbi and colleagues evaluated, in a phase II/III global randomized control trial, the impact of a combination of nivolumab and relatlimab, a lymphocyte activation gene 3 (LAG-3) inhibitor, in treatment-naïve metastatic or unresectable stage III melanoma as compared to nivolumab alone. Combination therapy demonstrated improved PFS in all sub-groups, including acral melanoma, with a median PFS of 10 months in the combination arm and a median PFS of 4.6 months in the nivolumab monotherapy arm [50].

Some investigators have suggested combining chemotherapy and immunotherapy in order to enhance the anti-tumor activity of the immunotherapy by inhibiting regulatory T cells. A multicenter retrospective study from China featuring 69 patients with metastatic melanoma, of which 28 were acral melanoma, showed that anti-PD-1 agents combined with temozolomide, an alkylating chemotherapy agent, resulted in a superior ORR (40% vs. 12.5%) as compared to anti-PD-1 agents alone [51]. This superiority translated to improved median PFS, as well as in the group who received temozolomide plus pembrolizumab as compared to patients who received pembrolizumab alone (9.8 months vs. 6.2 months) [51]. A recent single-center, phase II, non-randomized trial examining treatment-naïve unresectable stage III/IV acral melanoma treated with camrelizumab, an anti-PD-1 agent, apatinib, a vascular endothelial growth factor receptor 2 (VEGFR-2) inhibitor, and temozolomide demonstrated an ORR of 64%, with a DCR of 88% and median PFS of 18.4 months [52].

Lastly, regional and intralesional therapies are not well studied in acral melanomas. As these are often lesions of the extremities, there has been enthusiasm for the use of regional perfusion therapies for recurrent disease and/or unresectable disease. In a single study of 150 Chinese patients, of which 79% had acral melanoma with in-transit disease, the complete and partial response rates were 6% and 35%, respectively [53]. These rates are lower than those published in the United States and Australia, which notably had lower inclusion rates of acral melanomas. The OPTiM trial, which resulted in the FDA approval of talimogene laherparepvec (T-VEC) for patients with advanced melanomas, did not account for acral melanoma as a histologic subtype. Additionally, retrospective studies evaluating the efficacy of intralesional agents for cutaneous melanoma do not separately evaluate the response rates for the acral melanoma subtype outside of isolated case reports [54]. More studies identifying the specific efficacy of regional perfusion therapies and intralesional agents in acral melanoma are warranted.

## 3. Mucosal Melanoma

### 3.1. Epidemiology

Mucosal melanoma is a subtype of malignant melanoma that arises from the melanocytes within the mucosal membranes lining the respiratory, genitourinary, and gastrointestinal tracts of the body [11,55]. Mucosal melanomas account for 0.8–3.7% of all melanomas diagnosed annually [11]. While the incidence of cutaneous melanoma has increased in the United States, the incidence of mucosal melanoma appears to be stable over time [11,56]. Unlike in acral melanoma, there does not appear to be a racial difference in the incidence of mucosal melanoma [11,12].

### 3.2. Presentation and Histopathology

**Figure 2:**
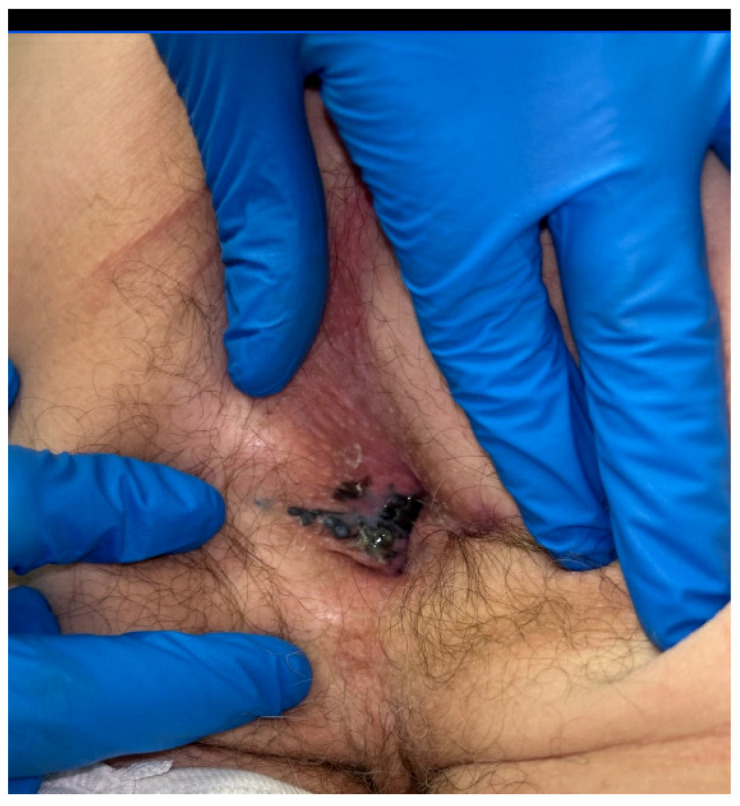
Mucosal melanoma of the anal canal.

Melanocytes originate from pluripotent neural crest cells and are able to migrate within the basal cell layer of the epithelium throughout the body, and as such, are present within all tissues to a varying degree. Even so, their presence does not translate to pigmentation within the tissue [57]. The presentation of mucosal melanomas is primarily driven by the disease site, but the presentation is often at a later stage due to the occult location of these lesions. Of all mucosal melanomas, 55% arise within the head and neck, 24% arise in the anorectum, 18% arise in the female genitourinary tract, and 3% arise in the male urinary tract [11]. Among those lesions that arise within the anorectum (Figure 2), one-third are present within the anal canal [55]. Among head and neck mucosal melanomas, the vast majority (60–80%) occur within sinonasal tissue [11]. Patients with mucosal melanomas are, on average, older than patients diagnosed with traditional cutaneous melanoma, with a median age of diagnosis of 70 years of age, and more likely to be female, particularly among those with genitourinary tumors [11]. Oral melanomas are more likely to arise among patients < 40 years of age [11]. These lesions have a high recurrence rate (23.5%) and an overall poor median progression-free survival of 14 months [58]. Lymph node metastases at the time of presentation are common, with 60% of anorectal and 20% of head and neck mucosal melanomas having clinically evident nodal disease at the time of diagnosis [57].

Mucosal melanomas arising from the head and neck locations more commonly occur in males and present with epistaxis or nasal obstruction if present within the sinonasal passages and cough, hemoptysis, or dusty-appearing sputum if in the main respiratory tract [55]. Lesions arising from the anorectum are most commonly present with bleeding, but 30% of these lesions will be amelanotic on examination [11]. Genitourinary lesions in women typically occur in those who are postmenopausal, and 15% of patients will have a family history of traditional cutaneous melanoma [11]. Vulvar melanoma is more prevalent than vaginal melanoma and represents the second most common malignancy of the vulva.

On histopathologic review, lesions present with a diverse arrangement of epithelioid, spindle, and plasmacytoid melanocytes aggregated into sheets or alveolar, nested groups [57]. AJCC TNM staging criteria are not routinely utilized in mucosal melanomas, and a standardized staging criterion has not been established [59]. Tumor thickness ≥ 2 mm is an independent predictor of worse prognosis; additionally, mitotic rate (>10 mitoses/mm^2^), ulceration, older age, male gender, and head and neck location have been shown to be independent risk factors for worse prognosis [11,55,57,60]. Cui and colleagues proposed a staging system for all anatomic sites of mucosal melanoma based on prospective data. In their study, mucosal melanoma was defined as stage I and II localized diseases based on tumor thickness of mucosal or submucosal versus muscularis or greater, and stage III disease was defined as a regional disease, but this criterion has not been widely accepted [61]. Unlike traditional cutaneous melanoma, genomic sequencing of mucosal melanoma tissue has shown its oncogenic development is UV sunlight-independent [62,63]. Unfortunately, risk factors and environmental drivers have not been identified as they pertain to the development of mucosal melanoma.

The overall tumor mutation burden in mucosal melanoma is quite low (approximately 10-fold less) when compared to traditional cutaneous melanoma (average somatic single nucleotide variant of 86,495 versus 8193) [64,65]. Mucosal melanomas are classified as having lower levels of somatic mutations, as well as lower levels of UV sunlight-induced mutations and fusion gene transcripts. Mutations that are noted in mucosal melanoma include neurofibromin 1 (NF1), KIT, and splicing factor 3b subunit 1 (SF3B1); NRAS and BRAF mutations occur at far lower rates than traditional cutaneous melanoma [12,21,66,67]. The rates of the latter mutations occur at up to 20%, with the highest prevalence among head and neck mucosal melanomas. Mutations within c-KIT and BRAF do not appear to correlate with survival [60].

### 3.3. Localized Disease

The standard of care for localized mucosal melanoma is wide local excision. However, given the varied location of these lesions, the extent of the procedure is often dictated by anatomical restraints. For example, sinonasal lesions can be excised either via an open surgical approach or through endoscopic resection. Wide margins can often be challenging given the location of lesions within the respiratory tract, and as such, adjuvant radiotherapy is often utilized to aid in minimizing recurrence rates, particularly given that recurrence rates are increased in margins <1 cm [55,57]. For anorectal melanoma confined to the gastrointestinal tract, surgical resection is warranted. Historically, melanomas arising from the anorectum were often managed with an abdominoperineal resection (APR), but more contemporary studies have not noted a survival benefit when compared to local excision [68,69]. Nilsson and Ragnarsson-Olding examined this using patients from the Swedish National Cancer Registry and noted a median OS of 14 months but no difference in survival when comparing these two procedures [70]. Even so, several studies have demonstrated that the primary prognostic factors associated with anorectal mucosal melanoma are the stage of disease and margin status following resection; this has historically resulted in the use of adjuvant radiation therapy to aid in local control of disease when negative margin excision is not possible [55,68]. Among localized genitourinary lesions, surgical resection with tumor-free margins is the standard of care [55,57]. Given the relative rarity of mucosal melanoma, there are limited data regarding the use of sentinel node biopsy in patients with mucosal melanoma. However, with the high propensity for metastatic disease in these patients, early staging of the regional lymph nodes may lead to earlier use of systemic therapy in these high-risk patients and should be considered [57,71,72].

### 3.4. Advanced Disease

Given the high rates of disease recurrence and propensity for metastatic disease among patients with mucosal melanomas, there has been significant interest in the availability of treatments for advanced mucosal melanoma. The median survival of metastatic mucosal melanoma is approximately 9 months [73]. A multicenter retrospective study comparing immunotherapy (either anti-PD-1 agents or anti-CTLA-4 agents) to systemic chemotherapy (primarily dacarbazine) showed an improved median OS for patients receiving immunotherapy as compared to conventional chemotherapy (16 months vs. 8.8 months) [74]. Additionally, Hamid and colleagues demonstrated an ORR of 22% and a median PFS of 2.8 months among patients with unresectable stage III/IV mucosal melanoma receiving pembrolizumab. Yet, the documented response rates in an initial study conducted by Postow and colleagues looking at the impact of ipilimumab monotherapy in patients with unresectable stage III/IV mucosal melanoma showed a meager 6.7% ORR, which included both complete and partial responses [75]. Shoushtari and colleagues performed a multicenter retrospective study in which 35 patients with mucosal melanoma were treated with anti-PD-1 agents and demonstrated an ORR of 23%, with a median PFS of 3.9 months [42]. The CheckMate-172 trial, a phase II multicenter study in patients with advanced melanoma who progressed on first-line therapy of ipilimumab, included 63 mucosal melanoma patients treated with nivolumab as their second-line therapy and noted a median OS of 11.5 months [46].

Given improved but modest response rates noted with the receipt of anti-PD-1 agents in patients with mucosal melanoma, additional studies have explored the impact of combination therapy in these patients. In a pooled analysis performed by D’Angelo and colleagues, a combination regimen of nivolumab plus ipilimumab, as compared to either agent in monotherapy, was examined in patients with advanced mucosal melanoma [76]. Combination immunotherapy was associated with a better ORR as compared to nivolumab monotherapy (37.1% vs. 23.3%) and ipilimumab monotherapy (37.1% vs. 8.3%), although inferior when compared to the ORR achieved among traditional cutaneous melanoma patients [76,77]. The higher side effect profile of ipilimumab has additionally led many investigators to question the utility of this agent in the management of mucosal melanoma. A retrospective cohort study from Dimitriou and colleagues compared the outcomes of the receipt of anti-PD-1 with or without ipilimumab in 545 mucosal melanoma patients and noted a statistically significant higher response rate only among naso-oral primary site lesions with combination therapy; for all other metrics, pembrolizumab monotherapy had similar outcomes, questioning the utility of the addition of ipilimumab [78].

The Relativity-047 phase II/III trial by Tawbi and colleagues included 51 patients with mucosal melanoma and compared relatlimab with nivolumab to nivolumab monotherapy in a sub-group analysis. There was no difference noted in median PFS between the combination therapy and monotherapy groups [50]. Ho and colleagues investigated the role of neoadjuvant anti-PD-1 with or without anti-CTLA-4 agents among patients with advanced mucosal melanoma and noted a median event-free survival of 9.2 months and an ORR of 47% [79].

In contrast to cutaneous melanoma, BRAF mutations are infrequent in mucosal melanoma. When BRAF V600E-mutant metastatic or unresectable mucosal melanoma (*N* = 12) patients were treated in a small study with BRAF inhibitors with or without a MEK inhibitor, the ORR was 20%, and DCR was 70% [40]. Though the rate of BRAF mutations is low in mucosal melanomas, the rate of c-KIT mutation is often higher than is present in traditional cutaneous melanoma. A phase II trial in China evaluating the efficacy of treatment with imatinib in 11 c-KIT-mutant or -amplified mucosal melanoma patients noted a median PFS of 3.5 months and a DCR of 53.5% [80]. Yet, in a multicenter, phase II trial of 17 c-KIT-mutant or -amplified mucosal melanoma patients, Hodi and colleagues reported an ORR of 64% with imatinib among those with c-KIT mutations but not amplification [81]. Lastly, a recent phase Ib, single-center trial examined the utility of toripalimab, a humanized immunoglobulin 4 monoclonal antibody against PD-1, in combination with axitinib in advanced, treatment-naïve mucosal melanoma. The investigators showed an ORR of 48.3% and a DCR of 86%, with a median PFS of 7.5 months [82]. Like acral melanoma, the reported response rates with systemic immune and targeted therapies in patients with mucosal melanoma are lower than those seen with cutaneous melanoma. However, there is a subset of patients that may have a meaningful response, and further studies attempting to identify these patients and/or the unique biological drivers behind their tumors are warranted.

## 4. Uveal Melanoma

### 4.1. Epidemiology

Uveal melanoma is a rare subtype of melanoma but represents the most common primary intraocular malignancy [83]. Uveal melanoma most commonly arises from choroidal melanocytes (90%) but can also arise from melanocytes in the ciliary body (6%) and iris (4%). These lesions occur at a higher incidence among non-Hispanic White, older (peak incidence at 70 years of age), and male patients [83,84]. The United States age-adjusted incidence of uveal melanoma is 4.6–5.1 per 1,000,000 people, yet the incidence approaches >8 per 1,000,000 people in Scandinavian countries [83,85].

Patient risk factors associated with the development of uveal melanoma include fair skin and inability to tan, light eye color, occulodermal melanocytosis (risk of developing uveal melanoma 1/400), choroidal nevi, and BRCA1 associated protein 1 (BAP1) mutations, a tumor suppressor gene present on chromosome 3p21.1 [83,84]. In fact, BAP1 mutations are noted in 47% of all uveal melanomas and 84% of patients with metastatic uveal melanoma, indicating a worse prognosis [86]. The Collaborative Ocular Melanoma Study (COMS) Study Group classifies choroidal nevi as melanocytic ocular lesions <5 mm in largest basal diameter and <1 mm in height [87]. Environmental factors associated with uveal melanoma include arc welding and occupations on aircraft among all uveal melanomas and UV sunlight exposure in anterior iris melanomas [84,87]. As UV sunlight is unable to penetrate the eye to reach the posterior eye and contribute to the development of choroidal melanoma, this factor is not implicated in the oncogenesis of these lesions [88]. Additionally, patients with cutaneous atypical nevi, those traditionally believed to be at increased risk for developing traditional cutaneous melanoma, have a 4.4–10.4 times increased risk of developing uveal melanoma.

### 4.2. Presentation and Histopathology

Patients with uveal melanoma are often (87%) symptomatic and present with painless loss or distortion of vision, though larger tumors can result in retinal detachment with photopsia [83]. If the lesion is anterior, there may be discoloration of the iris or episcleral injection that has been diagnosed as chronic conjunctivitis; if in the ciliary body, then patients can experience astigmatism due to intraocular lens displacement. Diagnosis is obtained through a detailed funduscopic evaluation with indirect ophthalmoscopy, and lesions under 3 mm may require enhanced depth imaging, such as optical coherence tomography [84]. On ophthalmoscopic examination, uveal melanomas may present with subretinal fluid, orange pigmentation, and thickness >2 mm [83,87]. Malignancy should be suspected for lesions that are dome-shaped as this suggests that there has been invasion through the Bruch’s membrane within the retina [83]. Increased pigmentation within the lesion and macrophage presence portents high-risk disease [87]. Additionally, B-scan ultrasonography can be useful in diagnosis; suspicious lesions show acoustic hollowing, choroidal excavation, and orbital shadowing [84]. Diagnosis is typically established via clinical examination, and imaging and biopsy are not routinely performed.

Fifty percent of patients with uveal melanoma develop metastatic disease, and eighty to ninety percent of these metastases arise within the liver due to hematogenous spread with a median OS of 10–13 months [83,89,90,91]. Uveal melanoma is generally clustered into two groups, class 1 and 2, based on their gene expression profile. Class 1 tumors rarely metastasize, while class 2 tumors have high rates of metastasis [92].

### 4.3. Localized Disease

Transpupillary thermotherapy (TTT) if choroidal, photodynamic therapy (PDT) if amelanotic and choroidal, and either iodine-125 plaque brachytherapy or proton beam radiotherapy if ciliary body or choroidal are local treatment strategies for uveal melanoma [83]. TTT can correlate to a high recurrence rate among uveal melanomas, as high as 20%, so ensuring it is utilized only on small (<3 mm) lesions away from the macula and optic nerve is necessary. Medium lesions are managed with either iodine-125 plaque brachytherapy or proton beam radiotherapy. Iodine-125 plaque brachytherapy is not recommended in lesions >10 mm due to the high risk of radiation retinopathy.

Surgical resection is often reserved for larger tumors or if there is a thought that radiotherapy could result in visual loss. Anterior tumors are amenable to selective resection or irido-cyclectomy. Enucleation has often been utilized for large, posterior uveal melanomas that cause poor visual distortion at the time of presentation or tumors with extraocular extension [83]. However, COMS compared the efficacy of enucleation versus iodine-125 plaque brachytherapy for medium-classification tumors and demonstrated that all-cause mortality at 5, 10, and 12 years was no different [93]. Additionally, COMS evaluated the utility of neoadjuvant radiotherapy in larger choroidal melanomas; when comparing patients who underwent surgical resection only as compared to neoadjuvant radiotherapy with iodine-125 plaque brachytherapy, there was no difference in survival [93]. Even so, surgical resection is a durable means of managing local disease, with local control of disease in approximately 90% of patients [90]. Among these patients, the overall 5-year survival was 72–84%. Regional lymph node metastatic spread is unlikely due to the absence of lymphatics within the eye, and thus, lymph node biopsy is not suggested.

### 4.4. Advanced Disease

Given the propensity for the development of metastatic disease, optimal treatment for advanced disease is paramount in uveal melanoma. While some studies have suggested a possible benefit from resection in a select group of patients, such as those with minimal tumor burden, the vast majority of patients have miliary disease throughout the remainder of the liver and, therefore, surgery is not routinely recommended for metastatic uveal melanoma to the liver [83]. Despite their significant role in advanced cutaneous melanoma, systemic immune and targeted therapies are less effective for uveal melanoma, with reported response rates between 5–10% [89].

In the CheckMate-172 trial, 63 (6%) had uveal melanoma, and the median OS following second-line therapy with nivolumab was 12.6 months [46]. PEMDAC is a multicenter phase II study in which a histone deacetylase (HDAC) inhibitor (entinostat) was concurrently used with pembrolizumab to upregulate the immune signaling of the tumor cells; median PFS and OS were 2.1 and 13.4 months, respectively [94]. In a phase II study examining the impact of combined immunotherapy with nivolumab and ipilimumab, the median OS was 19.1 months [95]. Dacarbazine, temozolomide, and fotemustine, traditional chemotherapy agents used to manage metastatic uveal melanoma, had ORRs ranging 0–10% in several randomized trials; even with the addition of the MEK inhibitor selumetininb, there was no improvement in OS [96,97,98]. Lastly, a phase III trial comparing tebentafusp, an antibody with an affinity-enhanced T-cell receptor fused to an anti-CD3 effector that functions to target glycoprotein 100-positive cells, to the investigator’s alternative choice (*N* = 378) noted that treatment with tebentafusp was associated with longer median OS (21.7 vs. 16 months) and PFS (3.3 vs. 2.9 months) in patients with HLA-A*02:01-positive metastatic uveal melanoma [99].

Given the relatively poor clinical response to systemic therapies and the high rate of miliary disease throughout the liver precluding surgical resection in patients with metastatic uveal melanoma, regional therapies have historically been utilized to aid in disease control, including liver-directed therapies such as yttrium-90 labeled microspheres, selective internal radiotherapy (SIRT), and transarterial chemoembolization, as well as chemosaturation through either isolated hepatic perfusion (IHP) or percutaneous hepatic perfusion (PHP) with melphalan [100,101]. Initial studies noted a median OS of 2.8–18.6 months following SIRT [102]. A retrospective study performed by Tulokas and colleagues noted that OS after SIRT decreased from 18.7 months if used as a primary therapy to only 7.8 months when used as a salvage therapy [103].

Over the last decade, hepatic perfusion techniques have been increasingly utilized in the management of liver-dominant metastatic uveal melanoma, predominately percutaneous hepatic perfusion (PHP). While previously approved in Europe, this therapy was recently approved in the United States by the FDA for the treatment of metastatic uveal melanoma to the liver. Disease-specific eligibility criteria include <50% of involved liver parenchyma in order to minimize post-perfusion hepatic failure and no, or limited, extrahepatic disease. Hepatic arterial access for perfusion is gained through the placement of an inflow catheter in the femoral artery. Isolation of the hepatic vasculature is achieved through veno-venous bypass with the utilization of a double-balloon catheter accessed via the femoral vein [89,100,101]. Initial studies demonstrated a median OS of 14.9–27.4 months and a median PFS of 8.1–11.1 months; the complete and partial response rates following the first PHP were 5–5.9% and 49–60%, respectively [104,105,106,107,108,109,110]. Importantly, while the rate of grade 3 and 4 events was 37.5%, with the majority being post-procedure coagulopathy, the 30-day mortality rate in these studies was only 1.1–1.8% [105,110,111].

In a phase II study, Meijer and colleagues reported a median OS of 19.1 months (*N* = 35) following PHP for liver-dominant metastatic uveal melanoma [95]. In a phase III trial of patients treated with PHP or the best alternative choice (control), the median PFS was 5.4 months for PHP as compared to 1.6 months in the control group; there was no difference in median OS (10.6 vs. 10.0 months), but this was attributed to a high turnover from the control group to the treatment group [112]. Initial results from the FOCUS (PHP-OCM-301/301A, NCT02678572) trial, a phase III trial comparing PHP to the investigator’s alternative choice (transarterial chemoembolization, ipilimumab, pembrolizumab, dacarbazine, etc.), demonstrated a prolonged PFS of 9 vs. 3.1 months in favor of PHP [113]. When compared to other regional therapies for metastatic uveal melanoma of the liver, a retrospective study compared outcomes in patients who underwent SIRT as compared to PHP and noted that PHP resulted in improved DCR (30% vs. 18%) and median OS (516 days vs. 300.5 days), suggesting that PHP is a more favorable treatment choice [114].

## 5. Desmoplastic Melanoma

### 5.1. Epidemiology

Desmoplastic melanoma is a rare subtype of cutaneous melanoma that is characterized by spindled melanocytes and dense collagenous stroma [115,116,117]. The incidence of desmoplastic melanoma is two per 1,000,000 persons, with an annual increased rate of 4.6% [115,116,117]. Desmoplastic melanoma, while rare, has an overall recurrence rate of 27.5%, with local recurrence rates ranging 3–19% [116,117,118]. Given the high rate of local recurrence, adjuvant radiotherapy is routinely used in certain high-risk desmoplastic melanoma to improve local control [119].

### 5.2. Diagnosis and Histopathology

**Figure 3:**
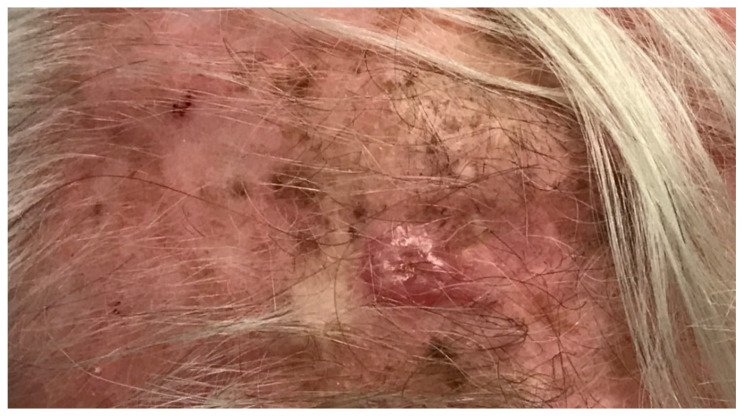
Desmoplastic melanoma of the scalp.

At presentation, desmoplastic melanomas may appear as a firm nodule, papule, or plaque that is often not pigmented (Figure 3) [116,117]. Approximately two-thirds of lesions will co-occur with either a melanoma in situ or lentigo maligna melanoma lesion [115]. Desmoplastic melanomas often present at a greater thickness. While these lesions are often mistaken for benign lesions, given their lack of pigment, it is possible the increased thickness at presentation is secondary to a delay in diagnosis. Of all desmoplastic lesions, half will occur in the head and neck. Patients with desmoplastic melanomas are often older (median age 60–70 years of age) and male, and similar to traditional cutaneous melanoma, lesions are often associated with chronic exposure to UV sunlight and sun damage [115].

Pathologic findings in desmoplastic melanoma include paucicellular proliferation of spindled melanocytes with collagenous or myxoid stroma and associated neurotropism [116,117,120]. Tumor thickness, mitotic rate, and the presence of lymph node metastases are predictive of melanoma-specific survival [118]. Desmoplastic lesions are classified as either pure or combined/mixed. Pure desmoplastic melanomas demonstrate >90% stromal fibrosis [118]. While they may present at increased thickness, the rate of lymph node metastasis is lower than other more common cutaneous melanoma subtypes [116,117]. Combined or mixed desmoplastic melanomas, classified as <90% stromal fibrosis, have a higher rate of lymph node metastases, distant recurrence, and overall worse prognosis than pure desmoplastic melanoma [115,118,121]. Hawkins and colleagues noted a five-year melanoma-specific death for pure desmoplastic melanomas at 11% and 31% for patients with combined/mixed desmoplastic melanomas [122]. Desmoplastic melanomas are often associated with a higher mutational burden and genomic profiles often associated with desmoplastic melanoma include inactivating mutations in NF1, promoter mutations in NFKBIE, and diverse activating mutations in the MAP kinase pathway, including those in ERBB2, MAP2K1, and MAP3K1 [21]. Similarly, the rate of BRAF V600K mutations is higher among desmoplastic melanomas and other melanomas associated with chronic sun-damaged skin [123].

### 5.3. Localized Disease Considerations

Similar to traditional cutaneous melanomas, the standard of care for localized desmoplastic melanoma remains wide excision with margins, as described by the NCCN guidelines. Yet, desmoplastic melanoma is often thicker, and as such, a greater proportion of these lesions will warrant excision with a 2 cm margin. Local recurrence is high among desmoplastic lesions compared to non-desmoplastic cutaneous melanoma [120]. Maurichi and colleagues compared pure and combined/mixed desmoplastic melanomas and noted that five-year mortality was higher for pure desmoplastic melanomas ≤2 mm thick when they were excised with a 1 cm margin as opposed to a 2 cm margin (40% vs. 14.8%); mortality outcomes were independent of excisional margins for combined/mixed lesions, likely due to the higher potential for metastatic disease [121]. Local recurrence incidence was increased in pure lesions that were managed with more narrow margins; this relationship did not translate to combined/mixed lesions. Chen and colleagues additionally examined the role of adjuvant radiotherapy following wide excision, with some margins as narrow as <5 mm, and concluded that the rate of local disease control was similar between those who did and did not receive radiotherapy, suggesting that radiotherapy can achieve disease control rates similar to adequate surgical excision [124]. Additionally, Guadagnolo and colleagues evaluated 130 desmoplastic melanoma patients and noted that the rate of local recurrence was significantly improved (7% vs. 24%) when comparing patients who underwent surgical excision followed by adjuvant radiotherapy to patients who underwent surgical excision alone [125].

While the role of wide local excision is defined, the utility of sentinel lymph node biopsy has been debated as the rate of lymph node metastases appears to be less than traditional cutaneous melanoma, estimated to be 4% in the literature. Yet, Pawlik and colleagues compared the positive sentinel lymph node rates of pure and combined/mixed desmoplastic melanomas to non-desmoplastic melanomas and noted that lymph node metastase rates were similar between mixed desmoplastic (15.8%) and non-desmoplastic (17.5%), but this rate was significantly lower among patients with pure desmoplastic melanomas (2.2%), suggesting that sentinel lymph node biopsy may play less of a role in the management of pure lesions [126]. Han and colleagues additionally demonstrated a significantly higher rate of sentinel lymph node metastases in combined/mixed (24.6%) desmoplastic melanomas as opposed to pure desmoplastic melanomas (9%) [127]. Additionally, Laeijendecker and colleagues noted that on institutional review of the Dutch Pathology Registry, combined/mixed histology was an independent risk factor for the presence of lymph node metastases [128]. As a 5% or greater risk of lymph node involvement is typically used to consider sentinel lymph node biopsy in patients with cutaneous melanoma, many centers will discuss and offer sentinel lymph node biopsy for patients with both pure and mixed desmoplastic melanoma.

### 5.4. Advanced Disease

Desmoplastic melanoma is known to carry a high tumor mutational burden, which correlates with high response rates to immune checkpoint inhibition. In a multicenter study analyzing 60 patients with advanced desmoplastic melanoma treated with anti-PD-1 therapy, ORR was 70%, and 32% of patients demonstrated a complete response. In accordance with prior studies, gene sequencing noted a high mutational burden and a higher proportion of PD-L1-positive cells within the tumor parenchyma [129].

## 6. Conclusions and Future Directions

Modern-era systemic immunotherapies and targeted therapies have improved overall survival for advanced melanomas and expanded the options available for patients over the past 15 years. Given the rarity of these discussed melanoma subtypes, the collective data on the efficacy of treatments for these lesions are often limited, and much of their management is extrapolated from larger trials in which they are underrepresented. Studies that have primarily included these patients have included retrospective reviews with smaller cohorts, limiting the strength of the data surrounding atypical melanoma subtypes. In these smaller or retrospective studies, it appears these systemic therapies are less effective in acral, mucosal, and uveal melanomas when compared to UV-induced cutaneous melanoma, while there is a suggestion of increased response rates and efficacy for desmoplastic melanoma.

There is ongoing investigation into the various treatment modalities that may be amenable for these rare melanoma subtypes. The recent SWOG S1801 trial (NCT03698019) resulted in significantly improved outcomes for high-risk, resectable melanoma when treated with neoadjuvant immunotherapy versus adjuvant therapy alone [6]. One specific benefit of neoadjuvant immunotherapy includes the opportunity to assess pathologic response in vivo and stop, or switch, therapy early for the progression of disease, which may be of particular interest in acral, mucosal, and uveal melanomas that exhibit lower response rates to immunotherapy. As such, a current trial examining apatinib and camrelizumab as a neoadjuvant regimen for patients with resectable acral melanoma is ongoing (NCT04331093). Farshidfar and colleagues identified that acral melanomas have a lower overall immune cell infiltrate as compared to traditional cutaneous melanoma, but of these infiltrates, PD-1, LAG-3, CTLA-4, VISTA, TIGIT, and ADORA2 were highly expressed, introducing new possible therapeutic targets [130]. Alternatively, the SWOG S1512 (NCT02775851) trial, a phase II trial, seeks to analyze the pathologic response rate among patients with stage II–III desmoplastic melanoma who receive neoadjuvant pembrolizumab. While this trial is ongoing, preliminary results have shown a pathologic response estimated to be more than twice that of the response noted among non-desmoplastic melanomas in prior trials; of 26 patients, the authors quote an 81% pCR. The RTN2 (NCT00975520) is a randomized trial comparing surgery alone and surgery plus adjuvant radiotherapy in neurotropic melanomas of the head and neck with the goal of evaluating the local recurrence rate. There is also ongoing work examining the benefit of PHP among metastatic uveal melanoma patients. CHOPIN is an ongoing single-center, phase Ib/phase II trial examining the combination of PHP with ipilimumab and nivolumab as the first-line therapy in liver-dominant metastatic uveal melanoma (NCT04283890). SCANDIUM-II (NCT04463368) is a similar study evaluating this question with IHP as opposed to PHP. More inclusion of these patients in large, randomized trials, along with a continued improvement in the understanding of the unique biological drivers associated with these rare melanoma subtypes, will ideally translate into improved therapies for these patients.
